# The Emerging Role of Extracellular Vesicle Derived From Neurons/Neurogliocytes in Central Nervous System Diseases: Novel Insights Into Ischemic Stroke

**DOI:** 10.3389/fphar.2022.890698

**Published:** 2022-04-26

**Authors:** Fan Li, Xiaokui Kang, Wenqiang Xin, Xin Li

**Affiliations:** ^1^ Department of Neurosurgery, Heji Hospital Affiliated Changzhi Medical College, Shanxi, China; ^2^ Department of Neurosurgery, Liaocheng People’s Hospital, Liaocheng, China; ^3^ Department of Neurosurgery, Tianjin Medical University General Hospital, Tianjin, China

**Keywords:** extracellular vesicles, oligodendrocytes, astrocytes, microglia, ischemic stroke, non-coding RNAs

## Abstract

Neurons and neurogliocytes (oligodendrocytes, astrocytes, and microglia) are essential for maintaining homeostasis of the microenvironment in the central nervous system (CNS). These cells have been shown to support cell-cell communication *via* multiple mechanisms, most recently by the release of extracellular vesicles (EVs). Since EVs carry a variety of cargoes of nucleic acids, lipids, and proteins and mediate intercellular communication, they have been the hotspot of diagnosis and treatment. The mechanisms underlying CNS disorders include angiogenesis, autophagy, apoptosis, cell death, and inflammation, and cell-EVs have been revealed to be involved in these pathological processes. Ischemic stroke is one of the most common causes of death and disability worldwide. It results in serious neurological and physical dysfunction and even leads to heavy economic and social burdens. Although a large number of researchers have reported that EVs derived from these cells play a vital role in regulating multiple pathological mechanisms in ischemic stroke, the specific interactional relationships and mechanisms between specific cell-EVs and stroke treatment have not been clearly described. This review aims to summarize the therapeutic effects and mechanisms of action of specific cell-EVs on ischemia. Additionally, this study emphasizes that these EVs are involved in stroke treatment by inhibiting and activating various signaling pathways such as ncRNAs, TGF-β1, and NF-κB.

## 1 Introduction

The neurovascular unit (NVU), which plays a vital role in neurological disorders, is a multifunctional and morphological entity composed of many cells and materials, including neurons, neurogliocytes, cells of brain vessels (pericytes, endothelial cells, smooth muscle cells), and the extracellular matrix (Potjewyd et al., 2018; Forró et al., 2021). Neurons and neurogliocytes, including oligodendrocytes, astrocytes, and microglia, are key players in maintaining the homeostasis of the microenvironment in the central nervous system (CNS). Under ischemic stroke conditions, hypoxia induces cell death and apoptosis and directly contributes to the release of pro- or anti-inflammatory cytokines and autophagy-related proteins from glia and neurons, leading to neuroprotection or neurotoxicity (Pan et al., 2021; Xin et al., 2021; Hou et al., 2022). Extracellular vesicles (EVs), present in multiple bodily fluids, are cell-derived nanoscale particles enclosed by a protein-rich lipid bilayer (Rodríguez and Vader, 2022). Based on their size, EVs can be classified into three types as follows (Saltarella et al., 2021): 1) apoptotic bodies (1,000–5,000 nm) are derived from apoptotic or necrotic cells that break up into multiple vesicles (Théry et al., 2009; Saltarella et al., 2021); 2) microvesicles (200–1,000 nm) are pinched off from the cell membrane directly (Théry et al., 2009; Saltarella et al., 2021); and 3) exosomes, the smallest EVs (30–150 nm), are produced *via* an active, energy-dependent, and organized process (Théry et al., 2009; Brinton et al., 2015; Saltarella et al., 2021). Although all classes of EVs and their heterogeneous subsets differ in their biogenesis, diameter, and cargo, owing to their natural composition, EVs have been suggested to exhibit a common feature of good biocompatibility and to transfer homing properties to specific cell types (Möller and Lobb, 2020; Roefs et al., 2020). Therefore, EVs are said to play an important role in intercellular communication as a new paracrine mediator (Möller and Lobb, 2020; Nagelkerke et al., 2021). All the aforementioned types of cells have been found to naturally secrete EVs under normal, physiological, and pathological conditions, owing to the dynamics of the cell membrane (Zhang et al., 2021a; Forró et al., 2021). A great deal of evidence has demonstrated that EVs derived from these cells exert biological functions by modulating specific aspects, such as participation in inflammatory reactions, cell migration, proliferation, apoptosis, and autophagy (Gao et al., 2020; Zhang et al., 2021a; Tallon et al., 2021). However, data on the precise mechanisms underlying such a therapeutic approach are limited. In this review, we summarize the effect of neuron/neurogliocyte-EVs in preclinical studies on treating neurological disorders by regulating different signaling pathways of cellular processes, such as cell apoptosis, inflammation, angiogenesis, and autophagy, and summarize the mutual mechanisms of the interaction between neuron/glial cell-EVs and ischemic stroke conditions.

## 2 The Characteristics and Biogenesis of ExtracellularVesicles

Despite the identification of well-recognized classes of cell-to-cell communication approaches, EVs have been a hot topic in the past several decades as an essential method for transferring cargo to short or long distances between cells (van Niel et al., 2018). Nevertheless, only a few people are aware of the discovery of EVs that can be traced back to the 19th century using a different nomenclature (Kaddour et al., 2021). In the 1840s, Gulliver’s first studied milky particles in the blood serum, which he called “the molecular base of the chyle,” with very small globules of active Brownian movement and a size ranging from −0.5 to 1 micron; this may have been the first encounter with EVs in the report (Gage and Fish, 1924). All eukaryotes can secrete EVs and exhibit a snapshot of the secreting cells, encapsulating active and specific biomolecules from the donor cell (Marzan and Stewart, 2021). Once EVs are produced in the extracellular space, these nanosized particles can be uptake by recipient cells, and in turn act as messengers and perform biological functions *via* the delivery of plenty of functional biomolecules including proteins, nucleic acids, lipids, and metabolites, into recipient cells, both near and far from the secreting cell (Palviainen et al., 2019; Marzan et al., 2021). Currently, there are three main subsets of EVs: exosomes, microvesicles, and apoptotic bodies. Exosomes are small EVs with a diameter of 30–150 nm and density of 1.13–1.19 g/ml (Yang et al., 2020). Exosome production is mainly classified into three stages: endocytosis, multivesicular body formation, and release. First, the plasma membranes start endocytosis, which is followed by the fusion of multiple intraluminal vesicles to produce endosomes. Second, the loading of bioactive molecules such as non-coding RNAs (ncRNAs), lipids, and proteins facilitates the formation of multivesicular bodies (van Niel et al., 2018). Finally, multivesicular bodies and plasma membranes are fused to achieve exosome release. Microvesicles with a diameter of 200–1,000 nm are slightly larger EVs generated by budding directly from the cell plasma membrane (Thietart and Rautou, 2020). This process involves lipid rearrangements concerning the asymmetry of the plasma membrane accelerated by membrane translocases, scramblases, and calpain. Conversely, apoptotic bodies, the largest among all types of EVs, are 1,000–5,000 nm in diameter, are secreted by dying cells, and are even more abundant than exosomes or microvesicles under specific conditions (Doyle and Wang, 2019; Battistelli and Falcieri, 2020). Many efforts have been made to identify the emerging role of exosomes and microvesicles in intercellular communication. However, there is little evidence on the value of apoptotic bodies in nanomedicine.

## 3 Extracellular Vesicles-Derived From Neurons/Neurogliocytes Regulate Cell Death and Apoptosis in Central Nervous System Diseases

Cell death is the consequence of multiple cellular processes that occur during neurological diseases, including mitochondrial dysfunction, protein aggregation, free radical generation, excitotoxicity, and inflammation (Salucci et al., 2021). Numerous studies have revealed that EVs derived from these cells are involved in cell death in neurological disorders. A previous study assessed the overall effects of exosomes derived from normoxic and hypoxic neurons on the survival and neuritogenesis of rat cortical neurons (Chiang et al., 2021). As presented by Chiang et al. (2021), hypoxic concentrated conditioned media, but not normoxic concentrated conditioned media, significantly reduced neuronal viability. They further pelleted exosomes from both normoxic and hypoxic concentrated conditioned media, and then examined the effects after administration of PBS and 25, 100, or 200 μg/ml exosomes in cultured cortical neurons using the CCK-8 assay. They noted a linear trend in the decrease in viability with increasing hypoxic exosome dose, revealing that hypoxic exosomes impair neuronal survival in a dose-dependent manner. However, low concentrations (25 and 100 μg/ml) of normoxic exosomes did not affect neuronal viability. To further evaluate the effect on axonal outgrowth, dissociated cortical neurons were treated with exosomes and subjected to immunostaining with an anti-Tau antibody. The results showed that exosomes derived from hypoxic neurons, but not EVs obtained from normoxic neurons, impaired both dendritic and axonal outgrowths of cultured cortical neurons (Chiang et al., 2021). Among the various programmed cell death pathways (Datta et al., 2020), apoptosis accounts for a large proportion of cell death through brain injury (Radak et al., 2017), which efficiently removes damaged cells from DNA damage or during development (Fan et al., 2020). Apoptosis plays an essential role in the homeostasis of normal tissues, and scientists have identified that EVs play essential roles in regulating cell apoptosis. Huang et al. (2020) indicated that not only hypoxic neuron-concentrated conditioned media but also EVs derived from hypoxic neurons exacerbate hypoxia-induced injury on transplanted mesenchymal stem cell viability, apoptosis, and oxidative stress *in vitro*. In addition to neuronal EVs, the effects of EVs derived from glial cells on cell death and apoptosis regulation have been studied more extensively. Bu et al. (2020) performed a series of *in vitro* experiments and demonstrated that astrocyte-derived exosomes promoted hypoxia-inhibited PC12 cell activity and suppressed cell apoptosis. Likewise, Chun et al. (2021) reported that astrocyte-derived EVs enhanced the survival and electrophysiological function of human cortical neurons. Notably, neuronal apoptosis was significantly increased upon treatment with conditioned medium from necroptotic astrocytes *via* EVs delivery (Chen et al., 2021). Furthermore, Casella et al. (2020) uncovered oligodendrocyte-derived EVs as an antigen-specific therapy for experimental autoimmune encephalomyelitis. This process was safe and restored immune tolerance by inducing apoptosis of autoreactive CD4^+^ T cells. Concerning the EVs derived from microglia, EV derived from both hypoxic and normoxic microglia can repress neuronal apoptosis and promote neuronal viability in hypoxic cortical neurons (Li et al., 2021a; Zhang et al., 2021a). To date, many studies have assessed the effect of EVs derived from neurons/glial cells in the regulation of apoptosis in CNS diseases (Song et al., 2019a; Xu et al., 2019; Bu et al., 2020; Casella et al., 2020; Datta Chaudhuri et al., 2020; Gao et al., 2020; Nogueras-Ortiz et al., 2020; Zhang et al., 2021a; Chen et al., 2021; Chun et al., 2021; Qi et al., 2021). The characteristics of these studies are summarized in Table 1 **.**

**TABLE 1:** Preclinical studies assessing the effect of EVs-derived from neurons/neurogliocytes on regulating cell death and apoptosis in CNS diseases.

Author, year	Country	Species	Model	Route	Cell source	Mechanism	Disease	Effect	References
Datta Chaudhuri et al. (2020)	United States	Rats	NA	co-incubation	AS	NA	NA	Inhibit	Datta Chaudhuri et al. (2020)
Song et al. (2019a)	China	Mice, Cells	MCAO	IV, co-incubation	BV2	miR-124	NA	Inhibit	Song et al. (2019a)
Xu et al. (2019)	China	Cells	OGD	co-incubation	Astrocytes	miR-92b-3p	Stroke	Inhibit	Xu et al. (2019)
Bu et al. (2020)	China	Rats, Cells	MCAO, OGD	IV, co-incubation	AS	miR-361/AMPK/mTOR/CTSB	Stroke	Inhibit	Bu et al. (2020)
Huang et al. (2020)	China	Cells	OGD	co-incubation	N2A	NA	Stroke	Promote	Huang et al. (2020)
Casella et al. (2020)	United States	Mice, Cells	MS	IV	OI	IL-10	MS	Inhibit	Casella et al. (2020)
Chen et al. (2021)	China	Rats, Cells	NA	co-incubation	AS	GDNF	NA	Promote	Chen et al. (2021)
Chiang et al. (2021)	Tianwan	Rats, Cells	OGD	co-incubation	neuron	microRNAs	Stroke	Promote	Chiang et al. (2021)
Chun et al. (2021)	United States	Cells	NA	co-incubation	AS	NA	NA	Inhibit	Chun et al. (2021)
Qi et al. (2021)	China	Rats, Cells	VD	IV	HNSCs	MIAT/miR-34b-5p/CALB1	VD	Inhibit	Qi et al. (2021)
Zhang et al. (2021a)	Germany	Mice, Cells	MCAO, OGD	IV	microglia	TGF-β/Smad2/3	Stroke	Inhibit	Zhang et al. (2021a)

NA, not available; AS, astrocytes; MCAO, middle cerebral artery occlusion; OGD, oxygen-glucose-deprivation; ECs, endothelial cells; OI, oligodendrocyte; MS, multiple sclerosis; GDNF, glial cell line-derived neurotrophic factor; VD, vascular dementia; HNSCs, hippocampal neural stem cells; AD, Alzheimer’s disease; Ref, Reference.

## 4 Extracellular Vesicle-Derived From Neurons/Neurogliocytes Regulate Autophagy in Central Nervous System Diseases

Autophagy is an evolutionarily conserved cellular mechanism (Wang et al., 2018), which is a program caused by the regulation of the internal conditions of cells (Sun et al., 2018), such as starvation, hypoxic nutrient deficiencies, and infection (Mo et al., 2020), leading to the degradation of toxic proteins, damaged organelles, and invading pathogens *via* the lysosomal pathway (Liu et al., 2020). On the one hand, autophagy can maintain cellular nerve homeostasis, since it is associated with degraded misfolded or nonfunctional proteins and damaged organelle, suggesting that it plays an essential housekeeping role in the CNS (Peker and Gozuacik, 2020). On the other hand, autophagy is also associated with the promotion of cell death. It is possible that excessive upregulation of autophagy and long-term autophagy eventually result in self-digestion or have harmful effects (Bar-Yosef et al., 2019). Abundant evidence indicates that autophagy and exosomes are inseparable. Autophagy plays a vital role in the synthesis and degradation of extracellular vesicles (EVs). It has been reported that autophagosomes not only have a strong ability to fuse with lysosomes, but also fuse with multivesicular bodies to form amphiphiles. The amphiphiles eventually fuse with lysosomes and dissolve the inner material of the intraluminal vesicles, resulting in a significant reduction in the release of exosomes. Taken together, these results suggest that autophagosome formation plays a key role in EVs secretion and transport (Xu et al., 2018). In addition, much direct evidence has verified that autophagy could be a therapeutic target in neurological treatment by using neuron/neurogliocyte-EVs. For example, in traumatic brain injury, Li et al. (2019) illustrated that neuronal EVs enriched with miR-21-5p can suppress neuronal autophagy induced by scratch injury, directly targeting the Rab11a 3′UTR region to reduce its translation, thus attenuating trauma-induced, autophagy-mediated nerve injury *in vitro*. Likewise, Liu et al. (2021a) found that M2 type microglia derived EVs could transfer miR-135a-5p into neurons to suppress the expression of thioredoxin-interacting protein, which in turn suppresses the activation of the nod-like receptor protein 3 inflammasome, thereby inhibiting neuronal autophagy induced by ischemia. To date, a vast number of studies have assessed the interaction between neuron/glial cell-EVs and autophagy in CNS diseases (Li et al., 2019; Pei et al., 2019; Guo et al., 2020; Pei et al., 2020; Zang et al., 2020; Liu et al., 2021a). The characteristics of these studies are summarized in Table 2 **.**

**TABLE 2:** Preclinical studies assessing the effect of EVs-derived from neurons/neurogliocytes on regulating autophagy in CNS diseases.

Author, year	Country	Species	Model	Route	Cell source	Mechanism	Disease	Effect	References
Pei et al. (2019)	China	Mice, Cells	MCAO, OGD	IV, co- incubation	AS	NA	stroke	Inhibit	Pei et al. (2019)
Pei et al. (2019)	China	Cells	OGD	co-incubation	AS	miR-190b	stroke	Inhibit	Pei et al. (2020)
Zang et al. (2020)	China	Mice, Cells	MCAO, OGD	SI, co-incubation	BV2	PDE1-B	stroke	NA	Zang et al. (2020)
Liu et al. (2021a)	China	Mice, Cells	MCAO, OGD	IV, co-incubation	microglia	miRNA-135a-5p/TXNIP/NLRP3	stroke	Inhibit	Liu et al. (2021a)
Guo et al. (2020)	China	Cells	NA	co-incubation	microglia	a-synuclein transmission	PD	NA	Guo et al. (2020)
Li et al. (2019)	China	Mice, Cells	TBI	co-culture, IV	BV2	miR-21	TBI	Inhibit	Li et al. (2019)

NA, not available; AS, astrocytes; MCAO, middle cerebral artery occlusion; OGD, oxygen-glucose-deprivation; SI, stereotaxic injection; IV, intravenous injection; α-syn, alpha-synuclein; TBI, traumatic brain injury; PD, Parkinson’s disease; Ref, reference.

## 5 Extracellular Vesicle-Derived From Neurons/Neurogliocytes Regulate Angiogenesis in Central Nervous System Diseases

Angiogenesis is the appearance of new microvessels that branch off from pre-existing vessels (Ruan et al., 2015). Hypoxic or insulted tissues can produce vascular endothelial growth factors, and angiogenesis begins along the concentration gradient of vascular endothelial growth factor in neonates (Carmeliet and Tessier-Lavigne, 2005). Angiogenesis plays a vital role in brain injury recovery following an injury because it promotes blood flow and metabolic nutrients to reach the injured regions to promote neural tissue repair by facilitating neurogenesis and synaptic initiation (Hatakeyama et al., 2020; Ma et al., 2021). To investigate whether microglial EVs regulate angiogenesis *in vitro*, Zhang et al. (2021a) showed that EVs derived from hypoxia-preconditioned microglia labeled with DiI were taken up by bEnd.3 endothelial cells. Further experiments concerning the therapeutic impact of these EVs against hypoxic injury of bEnd.3 cells were performed to evaluate cell viability and cytotoxicity *via* the MTT and LDH release assays, respectively. The results demonstrated that decreased cell viability and increased cytotoxicity after hypoxia were suppressed by these EVs (Zhang et al., 2021a). Moreover, these EVs can significantly promote bEnd.3 migration during hypoxia according to the scratch migration assay. Meanwhile, EVs derived from preconditioned microglia reversed this effect of impaired tube formation in bEnd.3 cells caused by hypoxia. Following the aforementioned *in vitro* findings, Zhang et al. (2021a) further indicated that EV administration induces angiogenesis and diminishes cell injury in the ischemic mouse hemispheres. Taken together, the evidence suggests that EVs derived from hypoxic microglia promote cell viability, migration, and angiogenesis in hypoxic injury. As such, the ability of EVs derived from neurons and other glial cells to regulate angiogenesis is unclear, and additional and reliable data are urgently needed.

## 6 Extracellular Vesicle-Derived From Neurons/Neurogliocytes Regulate Neuroinflammation in Central Nervous System Diseases

**FIGURE 1:**
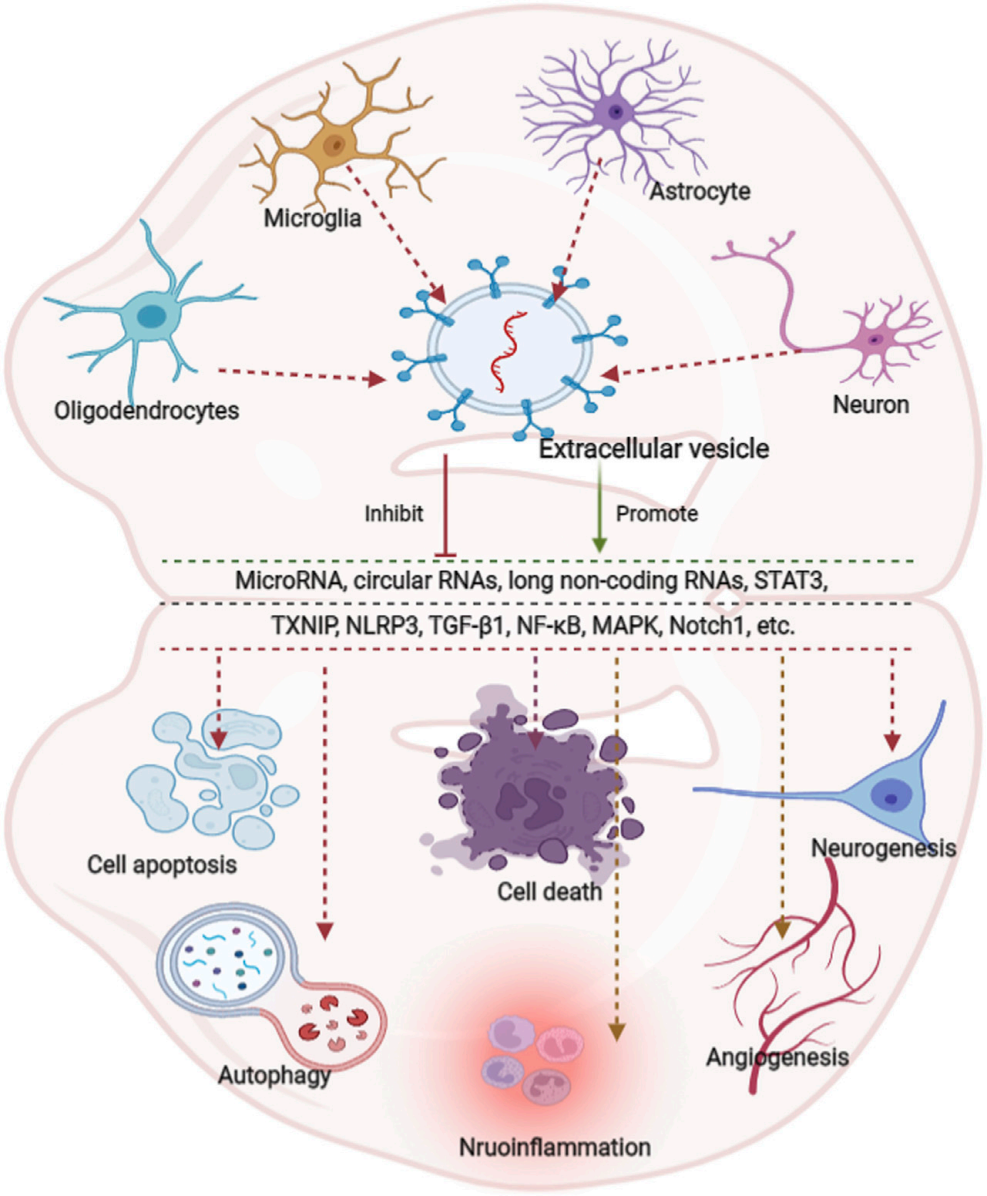
Overview of the effects of extracellular vesicle-derived from neurons/neurogliocytes on neurological recovery in central nervous system diseases. Predominantly extracellular derived from neurons/glial cells predominantly modulate autophagy, cell death, apoptosis, regeneration, and inflammation through various pathways. Glial cells are composed of astrocytes, microglia and oligodendrocytes. MicroRNAs (miRNAs) play key roles in various pathways.

Neuroinflammation is integral to the neurological pathophysiological process and results in damage to tissue homeostasis (DiSabato et al., 2016; Alawieh et al., 2018), involving acidosis, excitotoxicity, promotion of cytoplasmic Ca^2+^ concentrations, loss of glucose and oxygen, destruction of the blood-brain barrier, and damage to mitochondria (Xu et al., 2021; Chen et al., 2020a; Forrester et al., 2018). However, the inflammatory response is a double-edged sword after injury because it not only intensifies secondary injury to the brain, but also promotes the recovery of neurological function, thereby demonstrating that neuroinflammation is related to the pathogenesis and prognosis of CNS disorders (Zhang et al., 2021a). Several studies have revealed that various neuron/glial cell-EVs are involved in the regulation of inflammation and microglial activation in neurological diseases. Microglia serve as “brain-resident macrophages” that comprise approximately 10% of all the cells in the CNS (Fakhoury, 2018), the activation of microglia represents the first step of an inflammatory response, followed by the activation of other immune cells like neutrophils, T cells, natural killer cells, etc. (Xin et al., 2021; Iadecola and Anrather, 2011; Jin et al., 2010). Thus, the effects of microglial EVs on inflammation regulation have also been studied extensively. Casella et al. (2018) engineered a murine microglia cell line, BV-2 cell, to produce EVs enriched with the endogenous “eat me” signal Lactadherin on the surface to target phagocytes while overexpressing the anti-inflammatory cytokine IL-4. A single injection of these EVs into the cisterna magna upregulated anti-inflammatory markers, such as chitinase 3-like 3 and arginase-1, and significantly suppressed tissue damage in a mouse model of multiple sclerosis and experimental autoimmune encephalomyelitis. Likewise, overexpression of miR-124-3p in EVs derived from microglia following traumatic brain injury can reduce neuronal inflammation and contribute to neurite outgrowth by transferring these EVs into neurons (Huang et al., 2018). Similarly, BV2 cell-secreted EVs enriched with miR-711 could target and suppress Itpkb, thereby suppressing M1 microglial polarization and promoting M2 microglial polarization (Zhang et al., 2020). Besides that, the EVs derived from other glial cells, such as astrocyte and oligodendrocyte, also are involved in the regulation of inflammation (Casella et al., 2020; Li et al., 2020). The additional details are provided in Table 3 **.** EVs derived from glial cells might, therefore, contribute to suppressing inflammation and microglial activation (Casella et al., 2018; Huang et al., 2018; Zhang et al., 2020), information regarding the modulation of neuronal EVs in inflammation is scarce. An overview of how EVs derived from neurons/glial cells affect neurological recovery is shown in Figure 1 **.**

**TABLE 3:** Preclinical studies assessing the effect of EVs-derived from neurons/neurogliocytes on regulating neuroinflammation in CNS diseases.

Author, year	Country	Species	Model	Route	Cell source	Mechanism	Disease	Effect	References
Casella et al. (2018)	Italy	Mice, Cells	EAE	intrathecal injection-incubation	BV2	IL-4	MS	Inhibit	Casella et al. (2018)
Huang et al. (2018)	China	Mice, Cells	TBI, neuronal scratch-injury	IV, co-incubation	BV2	miR-124-3p	TBI	Inhibit	Huang et al. (2018)
Rojas et al. (2018)	United States	Mice, Cells	brain inflammation	IV	AS	DPTIP	NA	Inhibit	Rojas et al. (2018)
Chen et al. (2019)	China	Human	NA	NA	AS	IL-6	ALS	Inhibit	Chen et al. (2019)
Casella et al. (2020)	United States	Mice	EAE	IV	OI	IL-10	MS	Inhibit	Casella et al. (2020)
Datta Chaudhuri et al. (2020)	United States	Cells	NA	co-incubation	AS	ATP, IL-1β	NA	Inhibit	Datta Chaudhuri et al. (2020)
Li et al. (2020)	United States	Cells	NA	co-incubation	AS	CK1	AD	Inhibit	Li et al. (2020)
Zhang et al. (2020)	China	Mice, Cells	TBI	IV	BV2	miR-711	AD	Inhibit	Zhang et al. (2020)
Tallon et al. (2021)	United States	Mice, Cells	striatal IL1-β injection	IV	neurons, OI, microglia	nSMase2	NA	NA	Tallon et al. (2021)
Long et al. (2020)	China	Mice, Cells	LPS and TBI	IV, co-incubation	AS	miR-873a-5p	TBI	Inhibit	Long et al. (2020)

NA, not available; EAE, experimental autoimmune encephalomyelitis; MS, multiple sclerosis; TBI, traumatic brain injury; AS, astrocytes; DPTIP, 2,6-Dimethoxy-4-(5-Phenyl- 4-Thiophen-2-yl-1H-Imidazol-2-yl)-Phenol; ALS, amyotrophic lateral sclerosis; EAE, experimental autoimmune encephalomyelitis; OI, oligodendrocyte; AD, Alzheimer’s disease; nSMase2, neutral sphingomyelinase 2; Ref, reference.

## 7 The Role of Extracellular Vesicles-Derived From Neurons/Neurogliocytes in Ischemic Stroke

A comprehensive literature search of electronic databases, including PubMed, Cochrane Library, EMBASE, Web of Science, and China National Knowledge Infrastructure, was conducted from the inception of these databases until 28 February 2022. We retrieved studies assessing the effect of EV-derived from neurons/glial cells on ischemic stroke adopting the following keywords in accordance with Boolean logic: (“ischemic stroke” OR “middle cerebral artery occlusion” OR “MCAO” OR “ischemia”) and (“exosome” OR “EVs” OR “Extracellular vesicles” OR “microvesicles”) AND (“microglia” OR “neuron” OR “astrocyte” OR “oligodendrocyte”). In addition, all references from the included articles were manually checked to identify potential qualifying studies that were missed in the electronic search results. The process was repeated until no further studies would be obtained. A total of 29 studies were identified in this section (Frühbeis et al., 2013; Fröhlich et al., 2014; Guitart et al., 2016; Xin et al., 2017; Xu et al., 2017; Hira et al., 2018; Yang et al., 2018; Song et al., 2019a; Song et al., 2019b; Pei et al., 2019; Tian et al., 2019; Xu et al., 2019; Chen et al., 2020b; Bu et al., 2020; Frühbeis et al., 2020; Huang et al., 2020; Pei et al., 2020; Wu et al., 2020; Xie et al., 2020; Zang et al., 2020; Liu et al., 2021a; Yang et al., 2021a; Zhang et al., 2021a; Li et al., 2021b; Liu et al., 2021b; Zhang et al., 2021b; Chiang et al., 2021; Du et al., 2021; Raffaele et al., 2021), conducted from 2016 to 2021. The most extensively adopted species and associated stroke models are mice and middle cerebral artery occlusion, respectively. Among these 29 publications, 10 focused on microglia-EVs (Yang et al., 2018; Song et al., 2019a; Tian et al., 2019; Xie et al., 2020; Zang et al., 2020; Liu et al., 2021a; Zhang et al., 2021a; Li et al., 2021b; Zhang et al., 2021b; Raffaele et al., 2021), 11 focused on astrocyte-EVs (Guitart et al., 2016; Xin et al., 2017; Hira et al., 2018; Pei et al., 2019; Xu et al., 2019; Chen et al., 2020b; Bu et al., 2020; Pei et al., 2020; Wu et al., 2020; Liu et al., 2021b; Du et al., 2021), 3 focused on oligodendrocytes (Frühbeis et al., 2013; Fröhlich et al., 2014; Frühbeis et al., 2020) and 5 focused on neuron-EVs (Xu et al., 2017; Song et al., 2019b; Huang et al., 2020; Yang et al., 2021a; Chiang et al., 2021), demonstrating that it is sufficient to suggest that EVs derived from neurons/glial cells play an important role in regulating the progress and prognosis of ischemic stroke.

### 7.1 The Extracellular Vesicles Derived From Neurons

Accumulating evidence has demonstrated that neurons have the potential to release EVs from their somatodendritic compartments (Fauré et al., 2006; Lachenal et al., 2011) to regulate local synaptic plasticity, trans-synaptic communication, and post-stroke recovery. As mentioned, microglia are professional phagocytes that are, in part, beneficial due to their ability to reduce neuroinflammation *via* phagocytosis of dead neurons and neuronal debris (Sierra et al., 2013). Previous studies have revealed that neurons could inhibit microglial activation and promote M2-type microglial polarization, which in turn modulates neuronal survival during ischemic stroke (Norris et al., 2018; Pluvinage et al., 2019). As such, Yang et al. (2021a) indicated that these EV-derived miR-98 act as an intercellular signal mediating neuronal and microglial communication by suppressing platelet-activating factor receptor-mediated microglial phagocytosis during the recovery of neurological function induced by an ischemic stroke. Likewise, Song et al. (2019b) conducted an ischemic brain injury study that resulted in MCAO. They found that EVs derived from hypoxic neurons inhibited the expression of chemokine (C-X-C motif) ligand 1 (CXCL1) and inflammatory factors in astrocytes, suggesting that EVs derived from cortical neurons exert protective effects against neuroinflammation in astrocytes *via* downregulation of CXCL1. Additionally, Xu et al. (2017) demonstrated that these EVs could promote brain vascular integrity by modulating the levels of vascular endothelial cadherin binding to eukaryotic elongation factor 2 kinase. Herein, miR-132 serves as an intercellular signal that mediates the neural regulation of brain vascular integrity. Interestingly, as previously mentioned, EVs derived from hypoxic neurons, but not EVs obtained from normoxic neurons, impaired both dendritic, and axonal outgrowths of cultured cortical neurons (Chiang et al., 2021). Taken together, EVs derived from neurons have been shown to be collectively effective in the recovery of ischemic stroke patients; however, information regarding this aspect is scarce. Therefore, more evidence-based information is needed.

### 7.2 The Extracellular Vesicles Derived From Oligodendrocytes

Oligodendrocytes are neural tube-derived cells that have the ability to form myelin, a compact lamellar wrapping revealed on properly large fiber axons, thereby accelerating nerve conduction (Cohen, 2005). EVs derived from oligodendrocytes also mediate neuroprotection and promote neuronal homeostasis. Frühbeis et al. (2013) indicated that, triggered by neuronal signals, oligodendrocytes can release EVs derived from the multi-vesicular body which appears prevalent at periaxonal sites in myelinated nerves. In turn, neurons can internalize EVs derived from oligodendrocytes by endocytosis and recover EV cargo, thereby importing bioactive molecules. The supply of cultured neurons with EVs derived from oligodendrocytes increased neuronal viability under conditions of cell stress. Electrophysiological analysis using *in vitro* multi-electrode arrays also demonstrated an improved firing rate of neurons exposed to EVs derived from oligodendrocytes, and further western blot analysis showed increased activation of pro-survival signaling pathways (Fröhlich et al., 2014). These EVs can directly deliver antioxidant enzymes, such as catalase and superoxide dismutase 1. Additionally, Frühbeis et al. (2020) pointed out that oligodendrocyte-to-neuron EVs transfer promotes long-term neuronal maintenance by improving the metabolic state and promoting axonal transport in nutrient-deprived neurons, suggesting a novel mechanistic link between myelin diseases and secondary loss of axonal integrity. Oligodendrocyte-EVs might therefore contribute to supporting neurons; information regarding this aspect, however, is scarce.

### 7.3 The Extracellular Vesicles Derived From Microglia

Microglia are highly dynamic cells with the potential to transform their morphology from ramified to amoeboid and alter their phenotypes in response to ischemic insult (Xin et al., 2021). These opposing roles of microglia under ischemic conditions correlate with a distinct phenotype, as suggested by the proinflammatory M1 and anti-inflammatory M2 types (Xin et al., 2021). M1 phenotype microglia participate in exacerbating brain damage by secreting interleukin (IL)-6, IL-1β, nitric oxide, and tumor necrosis factor-α (Tang and Le, 2016; Cheng et al., 2019). M2 microglia remove necrotic tissue and stimulate tissue repair by releasing IL-4, IL-10, and transforming growth factor-β, thereby maintaining homeostasis (Ma et al., 2018; Zhang et al., 2018). Microglia have been shown to support cell-cell communication in the treatment of stroke *via* multiple mechanisms, most recently through the release of EVs. Correspondingly, a variety of pathological conditions also regulate microglial secretion, thereby affecting the main components of EVs. For example, inflammation induced by LPS can alter EV production in microglial cells and alter the cytokine levels and protein composition carried by EVs (Yang et al., 2018). Likewise, Zang et al. (2020) illustrated that the increase in autophagic flux using vinpocetine is related to the alteration of microglial EVs contents and properties to protect the survival and neurite structure of neurons against ischemic stroke. Taken together, microglia under different conditions may alter the cargo of microglial EVs, and thus have different functions. For example, M2 microglial EVs can reduce glial scar formation by repressing the expression of astrocyte proliferation gene signal transducer and activator of transcription 3 and glial fibrillary acidic protein. Similarly, M2 microglial EVs can also reduce neuronal autophagy and apoptosis, which further inhibits ischemic brain injury (Song et al., 2019a; Liu et al., 2021a). EVs secreted by microglial cell lines also play a significant role in the treatment of stroke. Tian et al. (2019) showed that IL-4-polarized microglial cells can ameliorate the injury induced by ischemic stroke by improving angiogenesis through the secretion of exosomes. Zhang et al. (2021b) reported that EVs derived from BV2 in the M2 phenotype were taken up by neurons and suppressed neuronal apoptosis in response to ischemic injury, which further reduced the infarct volume and behavioral deficits in MCAO mice. In addition, ischemic preconditioning can change the composition of EVs secreted by microglia, which plays a crucial role in the treatment of stroke. Xie et al. (2020) indicated that EVs derived from ischemia-preconditioned microglia regulate the TGF-β/Smad2/3 pathway to promote angiogenesis in a tube formation assay and neurological recovery in stroke mice. However, Zhang et al. (2021a) reported that microglial EVs inhibited brain microvascular endothelial cell proliferation and angiogenesis by impairing brain microvascular endothelial cell viability and integrity, as well as the loss of vascular formation. The EVs isolation process was conducted by ultracentrifugation, whereas Zhang et al. (2021a) adopted the method of PEG combined with ultracentrifugation. Different extraction methods of EVs may have different therapeutic effects on stroke owing to the alteration of the components of EVs. A series of studies have assessed the interaction between EVs derived from diverse microglia and stroke conditions. The main effects and primary mechanisms of these studies are summarized in Table 4, demonstrating that microglial EVs may offer a promising strategy for the treatment of ischemic stroke.

**TABLE 4:** Preclinical studies assessing the interaction between EVs-derived from microglia and ischemic stroke.

Author, year	Cell status	Main effects	Main mechanisms
Yang et al. (2018)	Normoxic	Inflammation alters cytokine levels and protein composition in microglial-EVs	IL-6, neuroinflammation
Song et al. (2019a)	M2 type	Attenuate ischemic brain injury and promote neuronal survival	miR-124
Tian et al. (2019)	IL-4-polarized BV2	Ameliorate the ischemia damage by promoting angiogenesis	miR-26a
Xie et al. (2020)	hypoxic	Aggravate ischemia induced brain microvascular endothelial cells damage and permeability	miR-424-5p/FGF2/STAT3
Zhang et al. (2021a)	hypoxia	Regulate inflammatory response, promote angiogenesis and repress apoptosis *in vivo* and vitro	TGF-β/Smad2/3
Li et al. (2021a)	M2 type	Glial scar formation *via* inhibiting astrocyte proliferation and migration	miR-124/STAT3
Liu et al. (2021a)	M2 type	Inhibit TXNIP and NLRP3, thereby reducing neuronal autophagy and ischemic brain injury	miR-135a-5p/TXNIP/NLRP3
Raffaele et al. (2021)	Normoxic	Improve post-stroke recovery by preventing immune cell senescence and favoring oligodendrogenesis	TNF
Zang et al. (2020)	Hypoxia	PDE1-B regulates autophagic flux and EVs biogenesis, in turn regulates neuronal survival under	PDE1-B
Zhang et al. (2021b)	M2 type BV2	Attenuate neuronal apoptosis and promote the recovery of neurological function	miR-137/Notch1

EVs, extracellular vesicles; TGF, transforming growth factor; N-SMase-2, neutral sphingomyelinase-2; STAT3, signal transducer and activator of transcription 3; TXNIP, thioredoxin-interacting protein; NLRP3, nod-like receptor protein 3; TNF, tumor necrosis factor; PDE, phosphodiesterase enzyme.

### 7.4 The Extracellular Vesicles Derived From Astrocyte

Astrocytes are the most numerous glial cell types in the mammalian CNS that regulate brain function, synaptic function, neuronal viability, integrity of the blood-brain barrier, and neural plasticity *via* interaction with neurons, and play essential roles in the progression of ischemia (Tahir et al., 2022). As such, astrocyte-derived EVs have been shown to improve neuronal survival, inhibit microglial inflammation, and promote post-stroke functional recovery. In terms of neuronal survival, these EVs can not only directly promote neuronal viability but also indirectly inhibit neuronal autophagy, inflammation, and apoptosis under hypoxic conditions. For instance, Pei et al. (2020) used the mouse hippocampal neuronal cell line HT-22 under oxygen and glucose deprivation (OGD) conditions to mimic ischemic injury. Confocal laser microscopy revealed that EVs isolated from primary astrocytes were taken up by the HT-22 cells. Further experiments demonstrated that these EVs promoted HT-22 cell vitality and apoptosis, as determined by the CCK-8 assay and TUNEL staining, respectively, and regulated the expression of inflammation-related factors (TNF-α, IL-6, and IL-1β) analyzed by ELISA, levels of apoptosis-related proteins (cleaved caspase-3, Bax, and Bcl-2), and autophagy-related proteins (Beclin-1, LC3-I/II, Atg7, and P62) by western blot. Similarly, Pei et al. (2019) and Chen et al. (2020b) revealed that astrocyte-derived EVs could suppress autophagy and ameliorate neuronal damage, and further findings showed the effect of EVs on the inhibition of OGD-induced neurons apoptosis *via* regulating autophagy (Pei et al., 2019). In addition to EVs derived from normoxic astrocytes, Xu et al. (2019) indicated that EVs released from ischemic preconditioned astrocytes ameliorated OGD-induced cell death and apoptosis. Concerning the regulation of inflammation and functional recovery, Liu et al. (2021b) established an OGD N9 microglial model and an MCAO rat model. These findings revealed that astrocyte-derived EVs inhibited OGD-induced injury and inflammation by regulating NLPR3, oxidative stress, and inflammatory factors (IL-1β and IL-18) in N9 microglia; reduced brain infarction; and improved MCAO rat neural functions. Additionally, a series of specialized *in vitro* experiments have confirmed that these EVs can alleviate nerve damage and promote functional recovery after stroke. Bu et al. (2020) showed that these EVs improved neurocognitive function by evaluating the neurological deficit score and reduced the cerebral infarct size by TTC staining and cerebral edema. The main effects and primary mechanisms of astrocytes-EVs on the treatment of ischemic stroke are summarized in Table 5 **.**

**TABLE 5:** Preclinical studies assessing the interaction between EVs-derived from astrocyte and ischemic stroke.

Author, year	Cell status	Main effects	Main mechanisms
Guitart et al. (2016)	Hypoxic	PrP-carrying EVs under ischemic stress protects against oxidative stress, hypoxia, ischemia, and hypoglycemia	PrP
Hira et al. (2018)	Sema3A	Increase prostaglandin D2 synthase and GSK-3β+, thus contribute to axonal outgrowth and functional recovery	GTPase 1/R-Ras/Akt/GSK-3β
Pei et al. (2019)	Normoxic	Suppress autophagy response, enhance neurons viability and ameliorate ischemic damage *in vivo* and vitro	LC3, P62
Xu et al. (2019)	Hypoxic	Protect neurons against OGD injury and elevate the cell viability	miR-92b-3p
Chen et al. (2020a)	Hypoxic	Suppress neuronal apoptosis and ameliorate neuronal damage *via* regulating autophagy *in vivo* and vitro	miR-7670-3p/SIRT1
Du et al. (2021)	Normoxic	Decrease BNIP2 expression, reduce oxidative stress, and inflammation in HIBD rats	miR-17-5p
Wu et al. (2020)	Normoxic	Downregulate the NF-κB/MAPK axis, thereby promote proliferation and inhibit apoptosis	miR-34c/NF-κB/MAPK/TLR7
Bu et al. (2020)	Normoxic	Increase cell activity and suppress cell apoptosis *in vitro* and alleviate nerve damage in rats	miR-36/AMPK/mTOR
Pei et al. (2020)	Normoxic	Attenuate neuronal apoptosis by suppressing autophagy	miR-190b/Atg7
Liu et al. (2021b)	Hypoxic	Inhibit inflammation *in vitro*, reduce brain infarction, and improve neural functions *in vivo*	miR-29a/NF‐κB/NLRP3

EVs, extracellular vesicles; PrP, prion protein; Sema3A, semaphorin 3A; HIBD, hypoxic-ischemic brain damage; TLR7, Toll-like receptor 7; MAPK, mitogen-activated protein kinase; SIRT1, sirtuin 1; CTSB, cathepsin B.

### 7.5 The Mechanism of Extracellular Vesicles Derived From Neurons/Glial Cells in the Effect on Regulating Stroke

#### 7.5.1 Non-Coding RNAs, Especially microRNAs, are the Key Players

Unlike messenger RNAs (mRNAs), which do not encode proteins, ncRNAs are ubiquitous throughout the human genome (Yang et al., 2021b). ncRNAs play an essential regulatory role in various biological processes such as cell proliferation, epigenetic modification, and cell apoptosis (Yang et al., 2021b). The top three most commonly described ncRNA molecules are miRNAs, long ncRNAs (lncRNAs), and circular RNAs (circRNAs) (Smith et al., 2021). miRNAs are a group of non-coding RNA molecules with a length of 19–25 nucleotides that participate in the regulation of gene expression after transcription by targeting the 3untranslated region of the target mRNA sequence and inhibiting mRNA levels (Navabi et al., 2022). lncRNAs are a class of single-stranded RNA molecules with more than 200 nucleotides that are important in molecular networks (Guo et al., 2022). CircRNAs are a class of non-coding RNAs with high stability and significant clinical relevance (Long et al., 2022). Intriguingly, as essential components, ncRNAs are selectively enriched in EVs, and ncRNAs loaded into EVs exert biological functions that modulate specific aspects of the onset and progression of ischemic stroke. Emerging evidence has demonstrated that a similar observation was also made for EV-derived ncRNAs derived from the aforementioned neurons/glial cells in ischemic stroke. For instance, Chen et al. (2020b) suggested that circSHOC2 expression was significantly upregulated in EVs released from ischemic-preconditioned astrocytes. Overexpression of circSHOC2 in neurons yielded the same protective effects as those from ischemic-preconditioned astrocyte-EVs *in vitro*, and similar results were also observed in MCAO mice by sponging miR-7670-3p, which regulates SIRT1 expression (Chen et al., 2020b). In addition to circRNAs, miRNAs are the most commonly reported miRNAs, as indicated in a series of publications. As mentioned above, M2 microglial EVs reduce glial scar formation and neuronal autophagy, mechanistically, M2 microglial EVs take effect *via* miR-124/STAT3 pathway and microRNA-135a-5p/TXNIP/NLRP3 axis, respectively. Meanwhile, neuronal EV-shuttled miRNA-181c-3p inhibited inflammation by downregulating CXCL1 in astrocytes in a rat model of ischemic brain injury (Song et al., 2019b).

#### 7.5.2 The Role of a Variety of Messenger RNAs Upon Extracellular Vesicle-Regulation in Stroke

**FIGURE 2:**
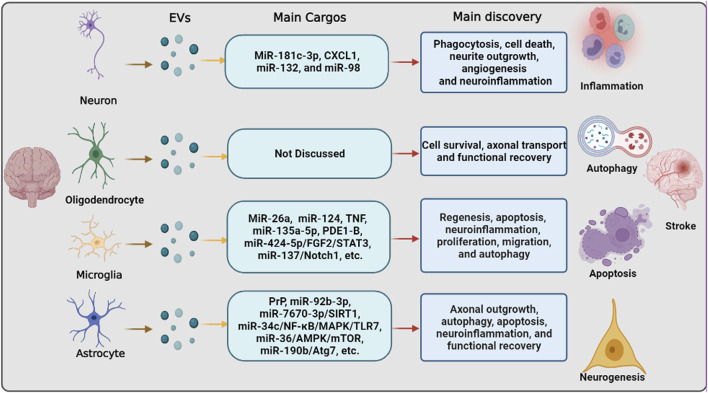
The pathways that are involved in extracellular vesicle derived from neurons/neurogliocytes to regulate stroke recovery. Neurons and various glial cells, including astrocytes, microglia, and oligodendrocytes, can regulate recipient cells by transferring extracellular vesicles to regulate various biological processes, including inflammation, autophagy, apoptosis, and neurogenesis, thereby regulating ischemic stroke progression and recovery.

mRNAs have been previously shown to have great potential for therapeutic applications in the treatment of ischemic stroke. Delivery systems for mRNAs, including lipid- and polymer-based carriers, have been developed to improve mRNA bioavailability. Among these systems, EVs are the most common carriers. For example, under OGD conditions, EVs derived from OGD-preconditioned primary microglia stimulated both angiogenesis and tube formation in bEnd.3 endothelial cells and repressed neuronal injury. Mechanistically, OGD induces upregulation of TGF-β1 in OGD-preconditioned microglia and EVs derived from non-hypoxic microglia or from different reoxygenation periods (24, 48, and 72 h) (Zhang et al., 2021a). Zhang et al. (2021a) used TGF-β1 siRNA to transfect microglia and obtained the corresponding EVs. Enriched TGF-β1 in EVs secreted from OGD-preconditioned microglia, but not microglia transfected with TGF-β1 siRNA, turned out to be a vital compound for the therapeutic potential of microglial EVs, affecting the Smad 2/3 pathway in both endothelial cells and neurons (Zhang et al., 2021a). This is in addition to the direct interaction between EVs and mRNA. Thus, neurons/glial cell-EVs can not only directly interact with mRNA but also indirectly affect mRNA by acting on miRNAs. Nuclear factor-kB (NF-κB) is present in almost all cell types and primarily serves as a transcription factor implicated in various biological processes (Barnabei et al., 2021). It has been shown to promote multiple pro-inflammatory mediators, and suppression of NF-κB signaling correlates with beneficial effects in ischemic stroke by EV-miRNAs. Liu et al. (2021b) reported that miR-29a in astrocyte-derived EVs inhibits brain ischemia-reperfusion injury by downregulating the NF-κB/NLRP3 axis. Wu et al. (2020) indicated that astrocytes-EVs transported miR-34c downregulates the NF-κB/MAPK axis and relieves neurological damage in ischemic stroke. Additionally, a great number of other mRNAs were revealed as the downstream of EV-miRNAs, such as Notch1 (Zhang et al., 2021b), AMPK/mTOR, (Bu et al., 2020), FGF2/STAT3 (Xie et al., 2020), etc. (Liu et al., 2021a). Besides that, there are, of course, many other pathways that are involved in EVs derived from neurons/glial cells to regulate stroke recovery as shown in Figure 2 **.**

## 8 Conclusion

EVs, ranging in size from 30 to 5,000 nm, secreted by different cell types, are surrounded by cell-segregated membrane complexes with a lipid bilayer and play an important role in the pathological and physiological environments of target cells by transferring a variety of cargo of nucleic acids, lipids, and proteins. Neurons and neurogliocytes, including oligodendrocytes, astrocytes, and microglia, play essential roles in maintaining homeostasis of the microenvironment in the CNS. These cells have been shown to support cell-cell communication *via* multiple mechanisms, most recently by the release of EVs, which further regulate various mechanisms underlying CNS disorders, including angiogenesis, autophagy, apoptosis, cell death, and inflammation. Ischemic stroke following cerebral artery occlusion is a major cause of chronic disability worldwide. Neuron/neurogliocyte-EVs are involved in the treatment of ischemic stroke under preclinical conditions. Among the mechanisms of these EVs in the treatment of stroke, ncRNAs play a key role, and they can also modulate directly or indirectly various mRNAs signaling pathways such as TGF-β1and NF-κB.
